# Pseudoexfoliation Syndrome and Sensorineural Hearing Loss

**Published:** 2011

**Authors:** Ramin Zojaji, Ali Alesheykh, Mohammad Reza Sedaghat, Kiamarz Navia, Morteza Mazloom Farsi Baf, Masoud Khaki, Aliasghar Raouf

**Affiliations:** 1 *Department of otorhinolaryngology, Islamic Azad University, Medical branch, Mashhad, Iran*; 2 *Department of ophthalmology, Islamic Azad University, Medical branch, Mashhad, Iran*; 3 *Department of ophthalmology,Mashhad University of Medical Sciences, Mashhad, Iran*; 4 *General physician,Islamic Azad University, Medical branch, Mashhad, Iran*; 5 *General physician,Islamic Azad University, Medical branch, Mashhad, Iran*; 6 *Manager of Education Development Center, Islamic Azad University, Mashhad branch, Mashhad, Iran*; 7 *Audiologist, Pejvak Audiology and Balance Center, Mashhad, Iran*

**Keywords:** Hearing loss, Pseudoexfoliation syndrome, Sensorineural deafness

## Abstract

**Introduction:**

Pseudoexfoliation syndrome (PXS) occurs due to the deposition of extracellular fibrillar materials on the anterior chamber of the eye. This syndrome has been considered to be part of a systemic disease with the potential involvement of the inner ear called sensoroneural hearing loss (SNHL). In this study, we aimed on evaluating SNHL within PXS patients in Iran to compare them with other international reports.

**Materials and Methods:**

In total, 33 patients with PXS and 33 age and sex matched controls were enrolled prospectively in a case-control study. Both groups underwent complete ophthalmologic and otorhinolaryngologic examinations and pure tone audiometry (PTA) testing. Six frequencies (0.25, 0.5, 1, 2, 3, 4 and 6 KHz) were evaluated for PTA in the same ethnic group in order to select the case and control individuals. Data were analyzed using t-test and chi-square test.

**Results:**

Forty-nine out of 66 ears (75.2%) in the PXS group and 27 ears (40.9%) in the control group had SNHL (P<0.001). No significant difference was found between the existence of exfoliative glaucoma (EXG) and SNHL in the PXS patients (P=0.768).

**Conclusion:**

Our results indicate a significant association between PXS and SNHL and may support the systemic nature of this disease.

## Introduction

Pseudoexfoliation syndrome (PXS) presents with the deposition of white and small extracellular fibrillar materials on the anterior chamber of the eye which results from the production and progressive collection of a compound glycoprotein agent with elastic fibers of basis (1,2) This can result in iridopathy, corneal endotheliopathy, vitreous loss, cataract and other lens involvements (). PXS is shown to be an infiltrative disorder which, therefore, tends to involve organs other than ocular tissue such as liver, kidneys, heart, lungs cardiovascular and cerebrovascular systems due to the accumulation of exfoliative materials in fibrovascular connective tissue septa of these organs, elastic and collagen fibers, fibroblasts and extracellular muscle cells (1,3 ,4). 

Besides and according to the same embryological origin of the ocular anterior segment and the basilar and tectorial membrane of the inner ear, concomitant involvement of the mentioned tissues have been demonstrated in some investigations (2,5 -7). Fibrillar material deposition in otic structures results in sensorineural hearing loss (SNHL) which is different from the age related hearing loss from the pathophysiological point of view. To date, studies with an acceptable number of audiometric frequencies and with the utilization of single ethnic groups in case and control sampling in order to evaluate the association between PXS and SNHL are lacking in our society (). Therefore, we conducted the present study on a more thorough evaluation of the association between PXS and SNHL in our population. 

## Materials and Methods

This comparative study was carried out on patients with PXS and age and sex matched controls from May 2009 to May 2010 in the ophthalmology clinic of our university affiliated institution, Aria Hospital, Islamic Azad University, Mashhad, Iran. 

All patients of 50 years of age and older with PXS whom were diagnosed ophthalmologically using slit lamp biomicroscopy, while mydriasis was induced, were considered for this study. Observation of exfoliative (fibrillar) material on the anterior lens capsule or on the corneal endothelium or on the pupillar margin, in one or both eyes was set to be the diagnostic criteria. Otolaryngologic investigation was performed on all patients and a present respiratory tract infection, history of otic surgery, acute or chronic otitis media, past or recent history of tympanic membrane perforation, ototoxic drug intake and regular noisy employment (work environment?) were considered as the exclusion criteria. According to the high prevalence of glaucoma in PXS, all patients were also examined for exfoliative glaucoma (EXG).

After one year, 33 patients with PXS were selected. Afterwards, 33 individuals of other ophthalmologic problems who had been ophthalmologically proven to have no pseudoexfoliative disorder were categorized into the control group. The mentioned exclusion criteria were considered for control individuals as well. 

All 66 patients and controls underwent pure tone audiometry (PTA). Hearing threshold level (HTL) was measured at 0.25, 0.5, 1, 2, 4 and 8 KHz of frequencies and hearing levels obtained from PTA were categorized into six groups: 0-20 decibels (dB), 21-40 dB, 41-55 dB, 56-70dB, 71-90 dB and more than 90 dB which were identified with normal hearing, mild, moderate, moderately severe, severe and profound hearing loss, respectively. 

The achieved data from both case and control groups was compared by SPSS software, version 15® (SPSS Inc., ). Baseline demographics and clinical characteristics were compared between the two groups using independent samples t-test, Chi-square and/or Fisher’s exact test. Statistical level of significance was set to be 0.05 and less. This study was approved by the institutional ethical committee and an informed consent was filled in by all patients prior to enrollment. 

## Results

In overall, the PXS group was consisted of 10 females and 23 males while the control group included 12 females and 21 males. The mean ages of the PXS and control groups were 72.2 yrs (range 50-88 yrs) and 72.8 yrs (range 53-85 yrs), respectively (*P*=0.939). 

The frequency of ophthalmic involvement in the PXS group is summarized in (Table 1). 

Among the 15 males and 4 females with bilateral PXS, only 1 patient in each group had unilateral glaucoma (both in the left eye) and apart from the 4 males and 1 female with bilateral glaucoma, the rest 10 males and 2 females had no kind of glaucoma. 

 The comparative result between the PXS and control group is summarized in (Table 2). 

As demonstrated, SNHL was present in 49 out of 66 ears (75.2%) in the PXS group compared to 27 ears (40.9%) in control group (*P*<0.001). The calculation of the different threshold levels included all the measured frequencies.

**Table 1:** Frequency of the PXS and EXG involved eyes in males and females

	**PXS**				**EXG**			
	Right eye	Left eye	Both eyes	Total	Right eye	Left eye	Both eyes	Total
**Male**	3	5	15	23	1	2	4	7
**Female**	3	3	4	10	2	2	1	5

PXS: pseudoexfoliation syndrome, EXG: exfoliative glaucoma

**Table 2:** Frequency of right/left/both ears hearing loss levels in all the measured frequencies in the PXS and control groups

Hearing level (dB)	Both ears			Left ear			Right ear		
	PXS	Control	Total	PXS	Control	Total	PXS	Control	Total
≤20	17 (25.8%)	39 (59.1%)	56 (42.4%)	9 (27.3%)	20 (60.6%)	29 (43.9%)	8 (24.2%)	19 (57.6%)	27 (40.9%)
21-40	39 (59.1%)	24 (36.4%)	63 (47.7%)	19 (57.6%)	12 (36.4%)	31 (47%)	20 (60.6%)	12 (36.4%)	32 (48.5%)
41-55	8 (12.1%)	3 (4.5%)	11 (8.3%)	4 (12.1%)	1 (3%)	5 (7.6%)	4 (12.1%)	2 (6.1%)	6 (9.1%)
56-70	0	0	0	0	0	0	0	0	0
71-90	2 (3%)	0	2 (1.5%)	1 (3%)	0	1 (1.5%)	1 (3%)	0	1 (1.5%)
≥90	0	0	0	0	0	0	0	0	0
Total	66	66	132	33	33	66	33	33	66

dB: decibels, PXS: pseudoexfoliation syndrome

In addition, no significant correlation was found between the side of ophthalmic (PXS) and otic (SNHL) involvement (*P*=0.847). This means that SNHL may occur in either left/right/both sides regardless of the PXS orientation. 

Moreover, our results support the lack of significant difference between Patients with and without EXG in the PXS group having 10 patients (83.3%) with EXG in comparison to 17 patients (81%) without EXG detected to have SNHL (*P*=0.865).

## Discussion

In this study, we found a significant relationship between PXS and SNHL in our population. According to the Published reports on the association of PXS and SNHL in the literature and especially those from the Middle Eastern countries (2,5-8), we attempted to evaluate if there is such concomitancy in Iran as well. Our comparative study consisted of two groups including the PXS patients group and a control group. The 33 patients in each group were examined for SNHL in both ears through air and bone conduction testing of which the results were compared together. EXG was also assessed in patients with PXS.

We found that hearing loss in PXS patients is significantly higher than the controls. In fact, 75.2% of ears in the PXS group had SNHL of various levels however, only 40.9% of the non-PXS patients were found to suffer from SNHL (*P*=0.001). Similarly, researchers have found significant difference between PXS and the control community as they discovered that 66.7% of their patients and 38.6% of their controls had SNHL (*P*<0.01). They compared 51 patients with PXS and 22 normal subjects whose mean ages were 67.5 and 61 years, respectively. Interestingly, in both studies a significant decrease in the number of patients with hearing loss in frequencies above 40 KHz but below 60 KHz (moderate hearing loss) was observed. Furthermore, this process continued and the number of patients declined more significantly at frequencies of 61 KHz and above (moderately severe hearing loss). This indicates that most of PXS patients in our study belonged to the normal hearing and mild hearing loss categories. The same distribution was observed in the studied community. In addition, the mean age of our population was higher than theirs. Hence, 4.7 years of age difference between our and their subjects may have resulted in the 8.5% higher SNHL occurrence in our PXS group; ironically, however, 11.8 years difference of age resulted in only 2.3% higher prevalence of SNHL in our control group. This emphasizes on the impact of PXS on SNHL progression (6). 

In 2008, Yazdani and colleagues performed a similar investigation on the Iranian population (). They compared 83 PXS patients with 83 age and sex matched individuals to define the difference between their hearing levels. SNHL was diagnosed in 88.4% of their PXS patients in contrast to 53.6% of their controls (*P*<0.001). The mean age of their patients and controls were 70.8 and 70.5 years, respectively. The difference between the age and prevalence percentage of patients with SNHL in the PXS category in our and their study was 1.4 years and -13.2%, respectively while the same corresponding amounts in the control societies were 2.3 and -12.7%, respectively. Similar to our work, both of their case and control populations included people of the same ethnic group; although they had solely used three frequencies (1, 2 and 3 MHz) for PTA (7). 

In addition to the above mentioned study, Cahill et al (6) as well as Shaban and Asfour (5) have compared HTL of PXS patients based on the International Standard (ISO 7029) median age association hearing loss at 1, 2 and 3 KHz of frequency. 73.7% and 87% of their PXS patients had a HTL over standard, respectively, which is similar to our results. 

In parallel, no significant relation was found between the side of ophthalmic and the side of otic involvement (*P*=0.847). This finding supports those achieved by Turacli and coworkers (). 

The only study in which tympanometric analysis has been performed as well as audiometry belongs to Detorakis et al from Greece () in 2008 they compared 54 and 48 patients in the two case and control groups, respectively. They concluded that tympanometric peak values are significantly lower in PXS patients and suggested that this phenomenon is due to the impairment in the elasticity of the middle ear structures in PXS patients which shows the systemic nature of this disease. 

Above all, EXG has also been studied in PXS patients. Similar to all published articles on this issue (5-8,10, 11), we found no significant difference in the frequencies in which SNHL occurs between patients with and without EXG (*P*=0.865). This denotes that patients with EXG do not suffer from a more severe disease than patients without EXG if the level of hearing loss has been considered as the index of severity in PXS. Obviously, however, our results do not criticize the reports suggesting that glaucoma on the basis of PXS has a more severe prognosis than in patients without PXS (). 

We attempted to select our patients by ophthalmological examination using slit lamp biomicroscopy. It is said that, however, in some cases of PXS, deposition of fibrillar materials cannot be found clinically and therefore histological investigation is required (). This could have resulted in some errors in sampling of the control group. Moreover, the sample size of our study was not large enough to increase the Power of the study efficiently either. 

To our knowledge this is the first study in which at the same time, 6 different frequencies (0.25, 0.5, 1, 2, 3, 4 and 6 KHz) were established for PTA in a population in which the case and control individuals were of the same race. 

The importance of ethnic groups in sampling of the case and control subjects is obvious but has not been yet fully studied. This point becomes more prominent when we have an international standard which has been set by the European and/or American associations. According to the comparative results of our study and others worldwide which have demonstrated a similar prevalence of SNHL within PXS patients, this standard system (ISO 7029) seems to be fully compatible with our country too. This eliminates the need for establishing control groups which require control measures of audiometric values in further Iranian studies. 

## Conclusion

Our results reconfirm the impact of PXS on the development of SNHL. The main reason of this association remains unclear although the infiltrative nature of PXS can still explain this concurrence. 

*Limitations:* The high mean age in our study participants can potentially affect the hearing threshold results by presbycusis. However, the PXS and control groups were age matched which can to some extent eliminate the probable bias in our conclusion. Moreover, the application of more detailed audiological analysis and examinations including otoacoustic emission, Bekesy audiometry or tinnitus matching and vestibular involvement evaluation are recommended in order to elucidate the pathology of this hearing disorder.

## References

[1] Ritch R, Schlotzer-Schrehardt U (2001). Exfoliation syndrome. Surv Ophthalmol.

[2] Turacli ME, Ozdemir FA, Tekeli O, Gokcan K, Gerceker M, Duruk K (2007). Sensorineural hearing loss in pseudoexfoliation. Can J Ophthalmol.

[3] Naumann GO, Schlotzer-Schrehardt U, Kuchle M (1998). Pseudoexfoliation syndrome for the comprehensive ophthalmologist. Intraocular and systemic manifestations. Ophthalmology.

[4] Schlotzer-Schrehardt U, Naumann GO (2006). Ocular and systemic pseudoexfoliation syndrome. Am J Ophthalmol.

[5] Shaban RI, Asfour WM (2004). Ocular pseudoexfoliation associated with hearing loss. Saudi Med J.

[6] Cahill M, Early A, Stack S, Blayney AW, Eustace P (2002). Pseudoexfoliation and sensorineural hearing loss. Eye (Lond).

[7] Yazdani S, Tousi A, Pakravan M, Faghihi AR (2008). Sensorineural hearing loss in pseudoexfoliation syndrome. Ophthalmology.

[8] Mohamed MM (2009). Detection of early glaucomatous damage in pseudoexfoliation syndrome by assessment of retinal nerve fiber layer thickness. Middle East Afr J Ophthalmol.

[9] Detorakis ET, Chrysochoou F, Paliobei V, Konstas AG, Daniilidis V, Balatsouras D (2008). Evaluation of the acoustic function in pseudoexfoliation syndrome and exfoliation glaucoma: Audiometric and tympanometric findings. Eur J Ophthalmol.

[10] Yuksel N, Keskin G, Altintas O, Karabas VL, Caglar Y (2006). Homocysteine levels in plasma and sensorineural hearing loss in patients with pseudoexfoliation syndrome. Eur J Ophthalmol.

[11] Shapiro A, Siglock TJ, Ritch R, Malinoff R (1997). Lack of association between hearing loss and glaucoma. Am J Otol.

[12] Prince AM, Streeten BW, Ritch R, Dark AJ, Sperling M (1987). Preclinical diagnosis of pseudoexfoliation syndrome. Arch Ophthalmol.

[13] Kivela T, Hietanen J, Uusitalo M (1997). Autopsy analysis of clinically unilateral exfoliation syndrome. Invest Ophthalmol Vis Sci.

